# A cross-sectional study of self-reported chemical-related sensitivity is associated with gene variants of drug-metabolizing enzymes

**DOI:** 10.1186/1476-069X-6-6

**Published:** 2007-02-10

**Authors:** Eckart Schnakenberg, Karl-Rainer Fabig, Martin Stanulla, Nils Strobl, Michael Lustig, Nathalie Fabig, Werner Schloot

**Affiliations:** 1 Institute for Pharmacogenetic and Genetic Disposition, Ostpassage 7, D-30853 Langenhagen, Germany; 2 Clinical Practice for Toxicology and Environmental Medicine, Immenhoeven 19, D-22417 Hamburg, Germany; 3 Center for Human Genetics and Genetic Counselling, University of Bremen, Leobenerstr. ZHG, D-28359 Bremen, Germany; 4 Children's Hospital, Pediatric Hematology and Oncology, Hannover Medical School, Carl-Neuberg-Str. 1, D-30625 Hannover, Germany

## Abstract

**Background:**

N-acetyltransferases (NAT) and glutathione S-transferases (GST) are involved in the metabolism of several ubiquitous chemical substances leading to the activation and detoxification of carcinogenic heterocyclic and aromatic amines. Since polymorphisms within these genes are described to influence the metabolism of ubiquitous chemicals, we conducted the present study to determine if individuals with self-reported chemical-related sensitivity differed from controls without self-reported chemical-related sensitivity with regard to the distribution of genotype frequencies of *NAT2*, *GSTM1*, *GSTT1*, and *GSTP1 *polymorphisms.

**Methods:**

Out of 800 subjects who answered a questionnaire of ten items with regard to their severity of chemical sensitivity 521 unrelated individuals agreed to participate in the study. Subsequently, genetic variants of the *NAT2*, *GSTM1*, *GSTT1*, and *GSTP1 *genes were analyzed.

**Results:**

The results show significant differences between individuals with and without self-reported chemical-related sensitivity with regard to the distribution of *NAT2*, *GSTM1*, and *GSTT1 *gene variants. Cases with self-reported chemical-related sensitivity were significantly more frequently *NAT2 *slow acetylators (controlled OR = 1.81, 95% CI = 1.27–2.59, *P *= 0.001). *GSTM1 *and *GSTT1 *genes were significantly more often homozygously deleted in those individuals reporting sensitivity to chemicals compared to controls (*GSTM1*: controlled OR 2.08, 95% CI = 1.46–2.96, *P *= 0.0001; *GSTT1*: controlled OR = 2.80, 95% CI = 1.65–4.75, *P *= 0.0001). Effects for *GSTP1 *gene variants were observed in conjunction with *GSTM1, GSTT1 *and *NAT2 *gene.

**Conclusion:**

The results from our study population show that individuals being slow acetylators and/or harbouring a homozygous *GSTM1 *and/or *GSTT1 *deletion reported chemical-related hypersensitivity more frequently.

## Background

Hypersensitivity against common environmental chemicals belongs to a complex of symptoms which are frequently reported by individuals suffering from a condition, variously referred to as multiple chemical sensitivity (MCS), chronic fatigue syndrome (CFS), or idiopathic environmental intolerance (IEI). In a position paper from 1999 the American College of Occupational and Environmental Medicine (ACOEM) stated that these terms refer to recurrent non-specific symptoms from multiple organ systems that the patients believe are provoked by exposure to low concentrations of chemical agents [1]. According to Cullen, the following criteria summarize the symptoms of these hypersensitivities: they are acquired after a specific health event in association with an environmental exposure, symptoms involve more than one organ system, symptoms recur and abate in response to predictable stimuli, symptoms are elicited by exposure to chemicals of diverse classes and modes of action, symptoms occur in response to very low levels of chemicals, and no widely available test of organ system function can explain the symptoms [2]. Nevertheless, the reasons for suffering from hypersensitivities to common environmental chemicals are unknown and it is supposed that genetic variants may influence individual response. In a recently published case-control study it was reported that polymorphisms of drug-metabolizing enzymes predisposed individuals to multiple chemical sensitivity [3]. However, since it is whether gene variants of drug-metabolizing enzymes are involved in the pathogenesis of idiopathic environmental intolerance, further data are necessary to characterise patients suffering from chemical hypersensitivity.

Glutathione S-transferases and N-acetyltransferases are biotransformation enzymes which are involved in the metabolism of ubiquitous chemical substances. Glutathione S-transferases catalyse glutathione-mediated reduction of exogenous and endogenous electrophiles. These enzymes have broad and overlapping substrate specificities and it has been hypothesized that allelic variants are associated with less effective detoxification of common chemical substances [4-6]. Analysis of DNA adducts and cytogenetic endpoints have indicated an increased susceptibility of glutathione S-transferase M1 and/or T1 (GSTM1, GSTT1) null genotype to genotoxicity of common low-dose chemicals [7-9]. For example, some chemicals cannot be conjugated by glutathione due to the deletion of GSTM1 gene. As a consequence of missing glutathione conjugation, chromosomal aberrations and sister chromatide exchange may be induced in lymphocytes exposed to a low dose of monoepoxybutene [10]. Glutathione S-transferases are involved in gene-environment interactions, may modify the individual predisposition to various diseases and were shown to influence the treatment response to drugs such as glucocorticoids and alkylating agents [11-16].

N-acetyltransferases are involved in the metabolism of arylamine and heterocyclic amines that are produced in industry, and found in cigarette smoke as well as the human diet. Bioactivation of arylamines and heterocyclic amines by N-hydroxylation is catalysed predominantly in the liver in various species and detoxification of arylamines is catalysed via N-acetylation [17]. The clearance of low-dose carcinogens have been described to be decreased in the genetically based slow-acetylator phenotype [18]. N-acetyltransferase 2 (NAT2) functional differences are explained by genetic variants within this intronless gene leading to the slow or rapid acetylator phenotype. Slow acetylators appear with a frequency of 50–60% in Caucasians. In a meta-analysis of 42 studies NAT2 has been identified to modulate susceptibility to colorectal cancer [19,20]. Other studies reported that acetylation by NAT2 has an impact on drug response [21-23]. Furthermore, it was shown to be a risk factor for individual susceptibility to various cancers like bladder cancer [24,25] and non-cancer diseases [26-28].

The lack of a generally accepted case definition for chemical hypersensitivity has delayed progress in this area. Miller & Prihoda developed a questionnaire called EESI (Environmental Exposure and Sensitivity Inventory) with self-rating scales to assess Symptom Severity, Chemical Inhalant Intolerances, Life Impact and Other Intolerances (e.g., foods, medications, alcohol) [29]. A sensitivity of 92% and specificity of 95% was achieved using scales of the questionnaire to differentiate cases from controls. Further investigation from an Asian population confirmed that findings from scales can be used for surveys and for diagnostic assessment of patients with idiopathic environmental intolerance [30].

This study was designed to evaluate chemical-related sensitivity to common ubiquitous substances in subjects with and without self-reported sensitivity and to analyze these findings in association with genetic variants of drug-metabolizing enzymes.

## Methods

### Study design

A modified questionnaire was used to collect information on individual chemical-related sensitivity from voluntary subjects (Table 1). This questionnaire included ten items associated with different ubiquitous chemicals. To assess reliability, the questionnaire was administered twice in 20 randomly selected volunteers, with the second administration occurring 7 days after the initial administration. The scores of the questionnaire of these subjects correlated at both dates. All 20 volunteers were identified as control or case due to a score of 10–20 or 21–30 points at both surveys. To assess validity, the questionnaire was compared to the part of the environmental exposure and sensitivity inventory (EESI, [29]) which asked for the same chemicals in our questionnaire. Our questionnaire used a rating scale of 1–3 in contrast to the rating scale of 0–10 in the EESI questionnaire. This standardized approach for measuring chemical intolerances was tested in 20 randomly selected volunteers. The scores of our questionnaire were in accordance with the scores of EESI. Subjects achieving a score of 10–20 or 21–30 points using our questionnaire corresponded to scores of 0–50 or 51–100 in the EESI questionnaire.

**Table 1:** Questionnaire of ten common chemicals.

Please indicate whether or not these odors or exposures would make you feel sick...	Not at all a problem	Moderate symptoms	Disabling symptoms
Diesel or gas engine exhaust			
Tobacco smoke			
Insecticide			
Gasoline			
Paint or paint thinner			
Cleaning products such as disinfectants, bleach, bathroom cleaners or floor cleaners			
Certain perfumes, air fresheners or other fragances			
Fresh tar or asphalt			
Nail polish, nail polish remover, or hair spray			
New furnishings such as new carpeting, a new soft plastic shower curtain or the interior of a new car			

In total, 800 randomly selected volunteers from a general practice in Hamburg, Germany, were asked to answer our questionnaire between September 1998 and April 2003. Out of these 800 subjects, 521 individuals agreed to participate in the study. Participants were instructed to use a rating scale that best corresponded to the severity of their sensitivity by checking a score of 1 to 3 points (not at all a problem, moderate symptoms, disabling symptoms) was to be marked by the subjects. A minimum of ten (all chemicals not a problem) and a maximum of 30 points (all chemicals disabling symptoms) were achievable. Participants were patients of a general practice with or without any disease for routine examination. None of the subjects was recruited according to the definition of Cullen [2]. Therefore, participants of our study were not assessed to one of the seven items which were proposed by Cullen for patients that suffer from multiple chemical sensitivity (MCS). Nevertheless, we can not exclude that patients with symptoms defined by Cullen are random participants of our study.

Subjects were divided into two groups according to the score achieved. Individuals with a score of > 20 were defined as sensitive to common chemicals (cases) while individuals with moderate or no symptoms were classified as non-sensitive (controls, ≤ 20 scores). The obtained scores were described as 'chemical-related sensitivity' scores (CRS). All individuals were of Caucasian origin with Caucasian parents living in the area of North Germany. The number of individuals born in the area of North Germany was equally distributed in cases and controls. The study conformed to good clinical practice guidelines and was carried out according to the guidelines of the Declaration of Helsinki. Written informed consent was obtained from each subject and the study was approved by the local ethic commission (Hannover, Germany). Participants were excluded from the study group of 800 individuals if they suffered from severe or chronic diseases like diabetes mellitus type I or II (n = 51) or oncological diseases (n = 13). Exclusion criteria were misusage of drugs or alcohol (n = 80) or misused exposition to any chemical (n = 25) which was assessed by asking the individual prior to fill the questionnaire or if they refused to participate into genotyping (n = 110). None of the 521 subjects refused participation in the study after genotyping.

### Genotyping

DNA was isolated from EDTA blood as described by Lahiri and Nürnberger or using the QIAamp DNA Blood Mini Kit [31]. After DNA extraction, the NAT2 gene was amplified as previously described [32]. The single nucleotide polymorphisms (SNPs) nt 481, nt 590, and nt 857 of the N-acetyltransferase 2 gene were analysed using RFLP/PCR or real-time PCR. The NAT2 nomenclature of the Arylamine N-Acetyltransferase Nomenclature Committee was used [33]. The genetic variants analysed in this study lead to a 4-allele model of the NAT2 gene which can predict the acetylator phenotype with an accuracy of more than 95% for slow and rapid acetylation [34]. Since the number of homozygous rapid acetylators (NAT2*4/*4) are small in Caucasians (approx. 5%) in contrast to Asian populations we did not differentiate between heterozygous and homozygous rapid acetylators.

The detection of homozygous deletions of the GSTM1 and/or GSTT1 was performed by multiplex-PCR as previously described [32]. Two gene variants within the GSTP1 gene leading to an amino acid exchange in exon 5 (I105V) and exon 6 (A114V) were analysed by means of PCR/RFLP. DNA (100 ng) was amplified (HotStarTaq, Qiagen, Germany) by 94°C (30 sec), 60°C (30 sec), 72°C (30 sec) and a final extension step at 72°C for 10 min. Primers were synthesised as described in the literature [35,36]. PCR fragments (each 25 μl) were digested with BsmA (5 units; I105V) and Cac8 (1,6 units; A114V) as described by the manufacturer (New England Biolabs, US). Electrophoresis was performed using a DNA LabChip system (Agilent Technologies, US). This protocol permits the identification of the GSTP1 alleles GSTP1*A (Ile105/Ala114), GSTP1*B (Val105/Ala114), GSTP1*C (Val105/Val114) and GSTP1*D (Ile105/Val114) according to Ali-Osman et al. [37].

### Statistical analysis

For descriptive purposes, frequencies of characteristics and common factors potentially affecting self-reported chemical sensitivity were obtained at the beginning of the analysis. To investigate the interrelationships between such factors, self-reported chemical sensitivity scores and *NAT2*, *GSTM1*, *GSTT1*, and *GSTP1 *genotypes, contingency tables and Spearman correlation coefficients were computed. The association of chemical-related sensitivity scores with *NAT2*, *GSTM1*, *GSTT1*, and *GSTP1 *genotypes was examined by use of univariate and multivariate unconditional logistic regression analysis to calculate odds ratios (OR) and their 95% confidence intervals (CI). *P *values of < 0.05 were considered statistically significant. Genotypes were used as categorical variables in these analyses. The SPSS statistical package (SPSS Inc., Chicago, IL) was used for computerized calculations.

## Results

In this study we applied a questionnaire asking for chemical-related sensitivity to ten common substances (Table 1) which was answered by 521 individuals seeking care at a single general medical practice between September 1998 and April 2003. This group of 521 individuals was then categorized in two groups at the median self-reported chemical-related sensitivity score (≤ 20 and > 20; see table 2). When investigating the association of factors potentially affecting the interrelationship of self-reported chemical sensitivity score and *NAT2*, *GSTM1*, *GSTT1*, and *GSTP1 *genotypes, we observed slight differences in the distribution of gender and smoking status between the two categories of self-reported chemical sensitivity scores. Individuals in the lower category with scores of ≤ 20 tended to show a smaller percentage of females and more current smokers. Age was differentially distributed between the two categories with no significant differences comparing the median age between the two groups (table 2).

**Table 2:** Characteristics of the entire study population and by CRS score (CRS ≤ 20; CRS > 20) in 521 subjects.

	All subjects n = 521	CRS≤ 20 n = 248	CRS > 20 n = 273	*P*^a^
Number of subjects (%)				

Gender				
Male	223 (42.8)	115 (46.4)	108 (39.6)	
Female	298 (57.2)	133 (53.6)	165 (60.4)	0.13
				
Age (years)				
0–9	2 (0.4)	2 (0.8)	-	
10–19	17 (3.3)	13 (5.2)	4 (1.5)	
20–29	32 (6.1)	21 (8.5)	11 (4.0)	
30–39	88 (16.9)	44 (17.7)	44 (16.1)	
40–49	105 (20.2)	42 (16.9)	63 (23.1)	
50–59	148 (28.4)	56 (22.6)	92 (33.7)	
60–69	90 (17.3)	45 (18.1)	45 (16.5)	
70–79	31 (6.0)	18 (7.3)	13 (4.8)	
80–89	6 (1.2)	5 (2.0)	1 (0.4)	
90–99	2 (0.4)	2 (0.8)	-	0.001^#^
				
Median age (range)	51.2 (7.5–98.0)	51.4 (13.9–84.6)	50.2 (7.5–98.0)	0.44
				
Smoking				
current	165 (31.7)	90 (36.3)	75 (27.5)	
former	23 (4.4)	11 (4.4)	12 (4.4)	
never	333 (63.9)	147 (59.3)	186 (68.1)	0.09

^#^calculated by using Kolmogorov-Smirnov test

Table 3 shows the distribution of *NAT2*, *GSTM1*, *GSTT1*, and *GSTP1 *genotypes by self-reported chemical-related sensitivity score (≤ 20 vs. > 20). The genotype distributions of each gene in the entire sample did not differ significantly from those predicted by the Hardy-Weinberg law. Table 3 also shows the association of *NAT2*, *GSTM1*, *GSTT1*, and *GSTP1 *genotypes with chemical-related sensitivity scores (≤ 20 vs. > 20). The risk of a score > 20 was significantly higher for study subjects carrying low-activity *NAT2 *alleles or showing homozygous deletions of *GSTM1 *and/or *GSTT1 *when compared to individuals with genotypes conferring higher enzyme activity. These results did not change in multivariate analyses when controlling for gender, age and smoking status. After stratification by gender the *NAT2 *results showed an increased odds ratio in women with a slow acetylator status in contrast to male subjects (Table 4). For *GSTP1*, no differences in the distribution of genotypes in the two categories were observed (table 3), neither in uni- nor in multivariate analysis.

**Table 3:** Association of the CRS score (CRS ≤ 20; CRS > 20) with NAT2, GSTM1, GSTP1 and GSTT1.

Gene	Genotype	CRS ≤ 20 n (%)	CRS > 20 n (%)	Univariate odds ratio (95% CI^a^)	Multivariate^b ^odds ratio (95% CI)	*P*^c^
		n = 248	n = 273			

*NAT2 fast*	*4/*4	15 (6.0)	13 (4.8)	1.00^d^	1.00^d^	
	*4/*5	68 (27.4)	49 (17.9)	0.83 (0.36–1.90)	0.83 (0.36–1.92)	0.668
	*4/*6	39 (15.7)	34 (12.5)	1.01 (0.42–2.41)	0.97 (0.40–2.33)	0.937
	*4/*7	3 (1.2)	5 (1.8)	1.92 (0.38–9.65)	1.88 (0.37–9.47)	0.446

*NAT2 slow*	*5/*5	49 (19.8)	61 (22.3)	1.44 (0.62–3.30)	1.51 (0.65–3.50)	0.339
	*5/*6	56 (22.6)	74 (27.1)	1.52 (0.67–3.46)	1.57 (0.69–3.60)	0.286
	*5/*7	4 (1.6)	5 (1.8)	1.44 (0.32–6.53)	1.46 (0.32–6.71)	0.627
	*6/*6	14 (5.6)	31 (11.4)	2.55 (0.96–6.77)	2.56 (0.95–6.89)	0.062
	*6/*7	-	1 (0.4)	n.c.^e^	n.c.^e^	-
	*7/*7	-	-	-	-	-

*NAT2 fast*	all	125 (50.4)	101 (37.0)	1.00^d^	1.00^d^	
*NAT2 slow*	all	123 (49.6)	172 (63.0)	1.73 (1.22–2.46)	1.81 (1.27–2.59)	0.001
*GSTM1*	*1/*1 or *0/*1	143 (57.7)	109 (39.9)	1.00^d^	1.00^d^	
	*0/*0	105 (42.3)	164 (60.1)	2.05 (1.44–2.91)	2.08 (1.46–2.96)	0.0001
*GSTT1*	*1/*1 or *0/*1	226 (91.1)	214 (78.4)	1.00^d^	1.00^d^	
	*0/*0	22 (8.9)	59 (21.6)	2.83 (1.68–4.78)	2.80 (1.65–4.75)	0.0001
*GSTP1*	*A/*A	106 (42.7)	117 (42.9)	1.00^d^	1.00^d^	
	*A/*B	82 (33.1)	101 (37.0)	1.12 (0.75–1.65)^e^	1.17 (0.79–1.75)^e^	0.433
	*A/*C or *B/*D	29 (11.7)	20 (7.3)			
	*A/*D	2 (0.8)	5 (1.8)			
	*B/*B	21 (8.5)	22 (8.1)			
	*B/*C	5 (2.0)	7 (2.6)			
	*C/*C	3 (1.2)	1 (0.4)			
	*C/*D	-	-			
	*D/*D	-	-			
	all except *A/*A	142 (57.3)	156 (57.1)	1.00 (0.70–1.41)	1.04 (0.73–1.48)	0.83

^a^confidence interval; ^b^adjusted for age (continous); gender; smoking (current; former; never); ^c^multivariate logistic regression; ^d^reference category; ^e^comprising all variant GSTP1 genotypes.

**Table 4:** Association of the CRS score (CRS ≤ 20; CRS > 20) with NAT2 genotype stratified by gender.

Gene	Genotype	CRS ≤ 20 n (%)	CRS > 20 n (%)	Univariate odds ratio (95% CI^a^)	Multivariate^b ^odds ratio (95% CI)	*P*^c^
		Females (n = 298)				

*NAT2 fast*	*4/*4	9 (6.8)	7 (4.2)	1.00^d^	1.00^d^	
	*4/*5	40 (30.1)	30 (18.2)	0.96 (0.32–2.88)	0.97 (0.32–2.91)	0.958
	*4/*6	24 (18.0)	22 (13.3)	1.18 (0.38–3.70)	1.18 (0.37–3.70)	0.780
	*4/*7	2 (1.5)	3 (1.8)	1.93 (0.25–14.89)	2.01 (0.26–15.56)	0.505

*NAT2 slow*	*5/*5	19 (14.3)	35 (21.2)	2.37 (0.76–7.37)	2.38 (0.76–7.42)	0.136
	*5/*6	29 (21.8)	43 (26.1)	1.91 (0.64–5.69)	1.93 (0.64–5.77)	0.241
	*5/*7	2 (1.5)	3 (1.8)	1.93 (0.25–14.88)	1.92 (0.25–14.84)	0.533
	*6/*6	8 (6.0)	21 (12.7)	3.38 (0.94–12.14)	3.44 (0.95–12.44)	0.059
	*6/*7	-	1 (0.6)	n.c.^e^	n.c.^e^	-
	*7/*7	-	-	-	-	-
*NAT2 fast*	all	75 (56.4)	62 (37.6)	1.00^d^	1.00^d^	
*NAT2 slow*	all	58 (43.6)	103 (62.4)	2.15 (1.35–3.42)	2.16 (1.35–3.44)	0.001

		Males (n = 223)				

*NAT2 fast*	*4/*4	6 (5.2)	6 (5.6)	1.00^d^	1.00^d^	
	*4/*5	28 (24.3)	19 (17.6)	0.68 (0.19–2.42)	0.68 (0.19–2.52)	0.568
	*4/*6	15 (13.0)	12 (11.1)	0.80 (0.20–3.13)	0.73 (0.18–2.95)	0.662
	*4/*7	1 (0.9)	2 (1.9)	2.00 (0.14–28.41)	1.45 (0.10–21.03)	0.786

*NAT2 slow*	*5/*5	30 (26.1)	26 (24.1)	0.87 (0.25–3.02)	0.93 (0.26–3.34)	0.905
	*5/*6	27 (23.5)	31 (28.7)	1.15 (0.33–3.98)	1.18 (0.33–4.24)	0.798
	*5/*7	2 (1.7)	2 (1.9)	1.00 (0.10–9.61)	0.95 (0.09–9.65)	0.963
	*6/*6	6 (5.2)	10 (9.3)	1.67 (0.37–7.61)	1.61 (0.33–7.76)	0.555
	*6/*7	-	-	-	-	-
	*7/*7	-	-	-	-	-
*NAT2 fast*	all	50 (43.5)	39 (36.1)	1.00^d^	1.00^d^	
*NAT2 slow*	all	65 (56.5)	69 (63.9)	1.36 (0.79–2.33)	1.45 (0.82–2.56)	0.199

**Figure 1:**
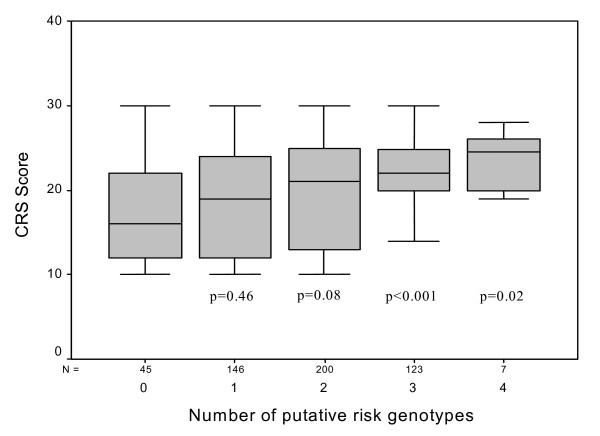
Self-reported chemical-related sensitity scores in dependance of number of putative risk genotypes (GSTM1 deletion, GSTT1 deletion, slow acetylation, GSTP1 variant genotypes) using Mann-Whitney U-Tests (p values were calculated by comparing putative risk genotypes with no-risk variants).

When we compared the number of gene variants between individuals with a chemical-related sensitivity score ≤ 20 vs. those with scores > 20, we observed an increasing chemical-related sensitivity score in association with the number of variant genotypes (Figure 1). Subjects with three putative risk genotypes (*GSTM1 *deletion and/or *GSTP1 *variant genotypes and/or *GSTT1 *deletion and/or *NAT2 *slow acetylator) harbour a significantly increased risk to report from chemical-related hypersensitivity than individuals without a gene variant. Interestingly, calculation of *GSTP1 *variant genotypes in combination with other variant genotypes showed an effect in regard to an additionnally increased CRS score (Figure 1).

## Discussion

In a meta-analysis using the database of the International Project on Genetic Susceptibility to Environmental Carcinogens (GSEC) the allele and genotype frequencies for many of the more commonly studied metabolic genes (CYP1A1, CYP2E1, CYP2D6, GSTM1, GSTT1, NAT2, GSTP1, and EPHX1) in the human population have been determined [38]. If we take together the genotype frequencies of cases and controls of our study we observed the same genotype frequency of GSTM1, GSTP1, GSTT1, and NAT2 as reported by [38]. These results indicate that the genotype frequencies in our study population did not deviate from published data and that the distribution of genotypes was not influenced by our study design. If individuals were divided in cases and controls using the scores from self-reported chemical-related sensitivity, we observed significant differences for distribution of genotype frequencies of GSTM1, GSTT1 and the NAT2 gene. Our results suggest that individuals with a deletion of GSTM1 or GSTT1 or slow acetyltators are at higher risk for developing self-reported chemical-related sensitivity.

The glutathione S-transferases are known to inactivate exogenous chemicals by glutathione conjugation. It is suggested that individuals with decreased glutathione conjugation are more prone to be unable to metabolize chemicals in the environment. It is also possible that both enzymes, GSTM1 and GSTT1, are metabolizing endogenous substrates less effectively due to gene deletion leading to an increased level of the parent compound. Although we did not differentiate between heterozygous and homozygous carriers of GSTM1 or GSTT1 by means of PCR, each of the homozygous deletions alone supported a direct role of the enzyme being associated with increased risk to self-reported chemical-related sensitivity. According to other authors, glutathione S-transferases play an important role in the detoxification of toxic chemicals. Nakajima et al. analysed GSTM1 genotype and total GST activity using 1-chloro-2,4-dinitrobenzene as a substrate and observed a greater GST activity in patients with the GSTM1 gene [39]. An interaction between GSTM1 genotypes and benzo [a]pyrene DNA adducts through air pollution in urban and rural areas was investigated in 120 healthy non-smoking residents indicating that the deletion of the GSTM1 gene may be an important step in the early onset of diseases [40]. It is also consistent with cases of our study group that homozygous GSTM1 deletion may be associated with an enhanced chemical-related sensitivity.

For GSTT1, there was a significant difference (OR: 2.80; p < 0.0001) between cases and controls in genotype frequency. This over-representation of cases with a homozygous GSTT1 deletion suggests that the GSTT1 enzyme plays an important role in glutathione conjugation of exogenous and/or endogenous substrates. This might result in cellular damage leading to an increased sensitivity if exposed to environmental chemicals. As described by other publications, individuals with a homozygous deletion of GSTT1 lack the possibility for enzymatic conjugation of environmental carcinogens such as 1,3-butadiene, ethylene oxide, epoxybutanes, methyl bromide, dichloromethane, and monohalomethanes [5-7]. In vitro experiments showed that the GSTT1 null genotype increased the sensitivity for sister chromatid exchange after exposure to diepoxybutane [41]. Genotoxic effects have been observed after exposure of lymphocytes to styrene or the metabolite styrene-7,8-oxide using a sister chromatid exchange assay [42,43]. These findings provide evidence that frequently used chemicals increase the susceptibility to develop chemical-related diseases. In addition, the GSTT1 null genotype conferred a 2.8-fold reduction in risk of relapse in childhood acute lymphoblastic leukemia indicating a more cytotoxic effect of chemotherapy [16]. In our study, we observed an increased chemical-related sensitivity in subjects with homozygous GSTT1 deletion leading to the assumption that the deletion of this gene augments the susceptibility to environmental chemicals.

No case-control differences were observed in genotype or allelic frequencies of GSTP1. Common chemicals are metabolized by GSTP1 and have been associated with risk to develop diseases like non-Hodgkin's lymphoma, hepatocellular and prostate carcinoma, as well as Alzheimer. Furthermore, Gilliland et al. reported a diesel exhaust particle enhancement in patients with GSTP1 Ile105Ile genotype [44]. However, we cannot exclude GSTP1 as a candidate gene for chemical-induced sensitivity since we did not analyse for promotor hypermethylation. Interestingly, the homoyzgous GSTP1*D/*D and the heterozygous GSTP1*C/*D genotype were not observed in cases and controls.

The GST enzymes, GSTM1, GSTT1 and GSTP1, are described to protect cells and organs from oxidative stress by conjugation of glutathione [46]. They detoxify a variety of electrophilic compounds, including oxidized lipid, DNA and catechol products generated by reactive oxygen species-induced damage to intracellular molecules. Therefore we can conclude that the deletion of GSTM1 and/or GSTT1 gene in individuals with chemical-related sensitivity lead to the loss of protection against oxidative stress. At the endpoint of this cellular process, individuals with chemical-related sensitivity may be more prone to symptoms like muscular pain, cardiovascular diseases, gastrointestinal disorders and several other symptoms that are described by patients suffering from MCS [2,3].

The results of our study show that cases were more frequently slow acetylators. The over-representation of the homozygous rapid acetylator genotype (NAT2*4/*4) reported by the study of McKeown-Eyssen et al. has been associated with the role of NAT2 in bioactivating arylamines to protein-binding metabolites [3]. The results of our study indicate that inactivation of arylamines through N-acetylation is an important mechanism. Wormhoudt et al. described that the slow acetylator genotype NAT2*6/*6 leads to a significant decreased acetylation capacity (11% vs. 100% compared to NAT2*4/*4) [45]. This genotype was observed more frequent in cases compared to controls in our study. Nevertheless, our results are not in contrast to the study of McKeown-Eyssen et al. since our study subjects were identified by a questionnaire asking for chemical hypersensitivity and not for symptoms of MCS. McKeown-Eyssen et al. drew up female patients from a larger study where participants were identified by symptoms described in six previously published MCS case definitions [2,3]. We also calculated our study subjects stratified by gender and observed that female slow acetylators were more prone to report chemical-related sensitivity than male subjects. We cannot exclude that some individuals of our study fit into one of the published MCS case definitions but since the results of the MCS study of McKeown-Eyssen et al. are symptoms-related, the results of our study are not comparable with those from McKeown-Eyssen's study. In addition, since the area of McKeown-Eyssen's study corresponds to the area where we did our study in regard to industrialisation, we suppose that slow acetylation and lack of glutathione conjugation is an important step to increase chemical-related sensitivity.

## Conclusion

In conclusion, we observed that individuals with self-reported chemical-related sensitivity were more frequent carriers of genetic variants of GSTM1, GSTT1 and NAT2. We believe that our results reflect the gene-environment associations of increased chemical-related sensitivity in individuals suffering from diseases like MCS, IEI or CFS but have to be reproduced in further studies to prove our observations.

## Abbreviations

CFS chronic fatigue syndrome

CRS chemical related sensitivity

CYP2D6 P450 cytochrome 2D6

EPHX microsomale epoxide hydrolase

GSTM1 glutathione S-transferase M1

GSTP1 glutathione S-transferase P1

GSTT1 glutathione S-transferase T1

IEI idiopathic environmental intolerance

MCS multiple chemical sensitivity

NAT2 N-acetyltransferase 2

OR odds ratio

PCR polymerase chain reaction

QEESI quick environmental exposure and sensitivity inventory

RFLP restriction fragment length polymorphism

SNP single nucleotide polymorphism

SPSS Statistical Package for the Social Sciences

## Competing interests

The author(s) declare that they have no competing interests.

## Authors' contributions

All authors were responsible for the concept of the study. ES and KRF coordinated the study. All authors were involved in sample collection, DNA preparation, genotyping and interpretation of the analyses. MSt and NS did the statistical analyses. MSt, KRF and ES drew-up the tables and prepared the manuscript with advice from the other authors.
